# Genomic attributes and characterization of novel exopolysaccharide-producing bacterium *Halomonas piscis* sp. nov. isolated from jeotgal

**DOI:** 10.3389/fmicb.2023.1303039

**Published:** 2023-12-14

**Authors:** Bora Kim, Ah-In Yang, Hae-In Joe, Ki Hyun Kim, Hanna Choe, Sung-Hong Joe, Min Ok Jun, Na-Ri Shin

**Affiliations:** ^1^Biological Resource Center, Korea Research Institute of Bioscience and Biotechnology, Jeongeup-si, Jeollabuk-do, Republic of Korea; ^2^Department of Biology, Kyung Hee University, Seoul, Republic of Korea; ^3^Department of Microbiology, Chonnam National University Medical School, Hwasun, Republic of Korea

**Keywords:** *Halomonas*, halophilic bacteria, jeotgal, exopolysaccharide, exopolysaccharide-producing bacteria

## Abstract

Halophilic bacterial strains, designated SG2L-4^T^, SB1M4, and SB2L-5, were isolated from jeotgal, a traditional Korean fermented food. Cells are Gram-stain-negative, aerobic, non-motile, rod-shaped, catalase-positive, and oxidase-negative. Phylogenetic analysis based on the 16S rRNA gene sequence revealed that strain SG2L-4^T^ is closely related to *Halomonas garicola* KACC 18117^T^ with a similarity of 96.2%. The complete genome sequence of strain SG2L-4^T^ was 3,227,066 bp in size, with a genomic G + C content of 63.3 mol%. The average nucleotide identity and digital DNA–DNA hybridization values between strain SG2L-4^T^ and *H. garicola* KACC 18117^T^ were 90.5 and 40.7%, respectively. The optimal growth conditions for strain SG2L-4^T^ were temperatures between 30 and 37°C, a pH value of 7, and the presence of 10% (w/v) NaCl. The polar lipids identified included diphosphatidylglycerol, phosphatidylethanolamine, phosphatidylglycerol, an unknown phospholipid, an unknown glycolipid, and an unknown polar lipid. The major cellular fatty acids were C_16:0_, summed features 8 (C_18:1_*ω*6*c* and/or C_18:1_*ω*7*c*), C_19:0_ cyclo *ω*8*c*, and summed features 3 (C_16:1_*ω*6*c* and/or C_16:1_*ω*7*c*). The predominant respiratory quinone was ubiquinone with nine isoprene units (Q-9). Based on the phenotypic, genotypic, and chemotaxonomic results, strain SG2L-4^T^ represents a novel species within the genus *Halomonas*, for which the name *Halomonas piscis* sp. nov. is proposed. The type strain is SG2L-4^T^ (=KCTC 92842^T^ = JCM 35929^T^). Functional annotation of the genome of strain SG2L-4^T^ confirmed the presence of exopolysaccharide synthesis protein (ExoD) and capsular polysaccharide-related genes. Strain SG2L-4^T^ also exhibited positive results in Molisch’s test, indicating the presence of extracellular carbohydrates and exopolysaccharides (EPS) production. These findings provide valuable insights into the EPS-producing capabilities of *H. piscis* sp. nov. isolated from jeotgal, contributing to understanding its potential roles in food and biotechnological applications.

## 1 Introduction

Jeotgal is a traditional Korean fermented food similar to kimchi, doenjang, and makgeolli. It is typically prepared by salting and fermenting seafood in brine for an extended period, resulting in a highly savory and pungent condiment that is used to enhance the flavor of various Korean dishes. Comprising primary ingredients such as eggs, gut, or whole body of fish or shellfish, jeotgal serves both as a standalone dish or sauce and as a flavor and fermentation enhancer when incorporated into other fermented foods like kimchi. The complex microbial community developed throughout jeotgal fermentation and its metabolites significantly affect the taste, flavor, and texture of the final product (Jung et al., 2018). The bacterial communities usually include the genera *Bacillus*, *Lactobacillus*, *Pseudomonas*, *Tetragenococcus*, *Sporosarcina*, *Virgibacillus*, and *Halomonas* (Guan et al., 2011; Song et al., 2018). Among these, *Halomonas* species are prominent halophilic bacteria that thrive under the high-salinity conditions characteristic of jeotgal, and are expected to contribute to promoting jeotgal fermentation.

*Halomonas*, a genus initially discovered by Vreeland in solar salterns, was reported with the description of *Halomonas elongate* (Vreeland et al., 1980). The genus *Halomonas* encompasses 121 species isolated from various saline environments such as saline lakes, salt flats, seawater, fermented fish sauce, and saline-alkali soils (Poli et al., 2007; Jiang et al., 2014; Li et al., 2022; Yoo et al., 2022; Lin et al., 2023). These bacteria, characterized by Gram-stain-negative, halophilic, rod-shaped, and catalase-positive, can grow under aerobic or facultative anaerobic conditions and exhibit a wide range of physiological and metabolic features.

One notable metabolic characteristic among *Halomonas* species is their capacity to produce exopolysaccharides (EPS). For instance, *H. rifensis* HK31^T^, *H. ventosae* Al12^T^, and *H. maura* S31^T^ have been reported to possess the capability of EPS production, as confirmed by the presence of EPS on the cell surfaces through scanning electron microscope (SEM) analysis (Bouchotroch et al., 2001; Martínez-Cánovas et al., 2004; Amjres et al., 2011). EPS are high-molecular-weight, long-chain carbohydrates secreted by microorganisms into their extracellular environment. Depending on their constituent sugars, these biopolymers can be categorized as homopolysaccharides, which consist of a single type of monosaccharide, or heteropolysaccharides, which consist of multiple monosaccharide types (Ruas-Madiedo et al., 2002). EPS play a pivotal role in various biological processes and have diverse applications (Poli et al., 2007; Joulak et al., 2019, 2022). In microbial communities, EPS contribute significantly to biofilm formation, providing structural integrity and protection against environmental stressors (Ghafoor et al., 2011). From an industrial perspective, microbial EPS have substantial commercial potential because of their natural occurrence, ease of extraction, low purification costs, and safety for both humans and the environment (Qi et al., 2022). For instance, *Lactobacillus fermentum* Lf2-extracted EPS improves yogurt texture and water-holding capacity, being a valuable natural ingredient with technological benefits (Ale et al., 2016). *H. saccharevitans* AB2, isolated from algae and rocks in the Red Sea, produces EPS with antimicrobial and antitumor activities (Abdrabo et al., 2018). Furthermore, administration of the EPS-enriched fraction obtained from the culture of *Bacillus subtilis* J92 improved intestinal barrier integrity and reduced inflammation in a dextran sulfate sodium-induced colitis mouse model, suggesting its potential as a pharmaceutical agent for inflammatory bowel disease management (Chung et al., 2021). These reports highlight the versatile applications of EPS across multiple fields, including food and pharmaceutical industries.

In this study, we have characterized three strains of the novel species *Halomonas piscis* sp. nov., isolated from jeotgal, using a polyphasic approach. Furthermore, we highlighted the potential uses of these novel strains by confirming the EPS production capability of *H. piscis*.

## 2 Materials and methods

### 2.1 Isolation and culture conditions of *Halomonas* strains

Jeotgal samples were purchased from a traditional market in Jeongeup-si, Jeollabuk-do, Republic of Korea (35°33′47.0”N, 126°51′19.0″E). Strains SG2L-4^T^, SB1M-4, and SB2L-5 were isolated from yellow corvina (*Collichthys lucidus*) jeotgal or mixed (shrimp, yellow corvina, pilchards, and anchovy) jeotgal and ripened for approximately 2 years with a final salinity of about 50% (w/v). The supernatants of the jeotgal samples were serially diluted 10^−1^ and 10^−2^-fold using sterile phosphate-buffered saline (Bioneer, Republic of Korea). One hundred microliters of diluted samples were spread onto Trypticase Soy Agar (TSA; Becton, Dickinson and Company, NJ, United States), Brain Heart Infusion Agar (Becton, Dickinson and Company), Gifu anaerobic medium (Nissui Pharmaceutical Co., Ltd., Japan), R2A Agar (Becton, Dickinson and Company), Marine Broth (Becton, Dickinson and Company), and *Lactobacilli* MRS Broth (Becton, Dickinson and Company) with 10% (w/v) NaCl, and then incubated at 25°C for 5 days. Colonies were randomly selected based on their morphological features and transferred to a fresh medium to purify a single colony. The isolated pure colonies underwent 16S rRNA gene sequence analysis and were preserved at −80°C in 40% glycerol stock. Reference strains for comparative analysis included three species of the genus *Halomonas*: *H. garicola* KACC 18117^T^ and *H. cibimaris* KACC 14932^T^ obtained from the Korean Agricultural Culture Collection (KACC), and *H. jeotgali* KCTC 22487^T^ obtained from the Korean Collection for Type Cultures (KCTC).

### 2.2 16S rRNA gene sequencing and identification

The 16S rRNA genes of strains SG2L-4^T^, SB1M-4, and SB2L-5 were amplified by colony polymerase chain reaction (PCR), following the conditions of Shin et al. (2018) with PCR pre-mix (BIOFACT, Republic of Korea) and two universal primer sets: 27F (5′-AGAGTTTGATCMTGGCTCAG-3′) and 1492R (5′-TACGGYTACCTTGTTACGACTT-3′) (Lane et al., 1985). The PCR products were subjected to Sanger sequencing on an ABI 3730XL analyzer (Thermo Fisher Scientific, MA, United States). The full-length sequences of the 16S rRNA genes were amplified and sequenced with primer sets 27F, 518F, 805R, and 1492R and assembled using SeqMan Pro software (DNAstar, WI, United States) (Lane et al., 1985; Stackebrandt and Goodfellow, 1991). Almost complete 16S rRNA gene sequences of strains SG2L-4^T^ (1,450 bp), SB1M-4 (1,480 bp), and SB2L-5 (1,429 bp) were obtained and closely related type strains were confirmed using the EzBioCloud database.^1^ Pairwise similarity values were determined with the EzBioCloud pairwise nucleotide sequence alignment tool.^2^ Similarities between strains were represented in a heatmap generated using the Heatmapper server.^3^ Alignment of 16S rRNA gene sequences was performed using the CLUSTAL W tool in BioEdit (Hall et al., 2011). Phylogenetic trees were constructed using neighbor-joining (NJ), maximum-parsimony (MP), and maximum-likelihood (ML) algorithms with 1,000 bootstrap replications using MEGA 11 software (Tamura et al., 2021). Evolutionary distances were calculated using the Tamura-Nei model.

### 2.3 Genomic features

Whole-genome sequencing was performed at a Macrogen facility (Macrogen, Republic of Korea). Genomic DNA was extracted from strain SG2L-4^T^ and sequenced using the PacBio Sequel system and Illumina HiSeq sequencing platform. The PacBio reads were used for *de novo* assembly via Microbial Genome Assembly application. Reads were mapped against assembled contigs, and polishing was performed using Racon, resulting in a consensus sequence of higher quality. Illumina reads were applied after assembly to enhance the genome sequence’s accuracy, utilizing Pilon software for polishing and correcting errors as well as filling gaps. The genomic DNA of strains SB1M-4, SB2L-5, and *H. garicola* KACC 18117^T^ was employed for draft genome sequencing using the Illumina HiSeq platform. The G + C content was determined using genomic DNA sequencing data. A phylogenomic tree was constructed using the Up-To-data Bacterial Core Genes pipeline and FastTree program (Na et al., 2018). Genome sequences of species closely related to the novel strains were obtained from the publicly available NCBI GenBank website. The average nucleotide identity (ANI) and digital DNA–DNA hybridization (dDDH) values between the isolated genomes and closely related strains were calculated using the OrthoANI tool^4^ (Lee et al., 2016) and Genome-to-Genome Distance Calculator v3.0, on the DSMZ server^5^ (Meier-Kolthoff et al., 2013), respectively. Functional annotation of the genome sequences was performed using the Rapid Annotation using Subsystem Technology (RAST) server^6^ (Aziz et al., 2008). A circular map of the annotated genome was generated using the CGView online service (Stothard et al., 2019).

### 2.4 Growth test

To determine their growth range and optimal growth conditions, the growth rates of strains SG2L-4^T^, SB1M-4, and SB2L-5 were tested at various temperatures, salinities, and pH values. The optimal temperature and pH values were determined by growing the isolates in tryptic soy broth (TSB; Becton, Dickinson and Company) containing 10% (w/v) NaCl at 4, 10, 15, 20, 25, 30, 37, 45, 55, and 65°C and pH values between 4 and 11 with 1 pH unit intervals. The pH was adjusted using 6 N HCl, 10 N NaOH, and buffers of 2-morpholino-ethanesulfonic acid (MES; pH 4–6), 4-(2-hydroxyethyl)piperazine-1-ethanesulfonic acid (HEPES; pH 7–9), or 3-(cyclohexylamino)-2-hydroxy-1-propanesulfonic acid (CAPSO; pH 10–11) at a concentration of 10 mM. The optimal growth salinity was determined by adding NaCl at concentrations of 0, 1, 2, 3, 4, 5, 7, 10, 15, 20, 25, 28, and 30% (w/v) to TSB without NaCl. The optical density at 600 nm (OD_600_) was measured using a Multiskan SkyHigh microplate spectrophotometer (Thermo Fisher Scientific) after 1, 2, and 7 days of incubation.

### 2.5 Morphological and physiological test

Phenotypic characterizations of strains SG2L-4^T^, SB1M-4, and SB2L-5, and reference strains were determined by culturing them in TSA containing 10% (w/v) NaCl at 30°C for 2 days. Cell morphology and the presence of flagella were determined using a JEOL JSM 7600F field emission SEM (JEOL Ltd., Japan) after fixation with 3% (w/v) glutaraldehyde at 4°C for 24 h (Li et al., 2019). A motility test was performed by culturing cells in 0.3% (w/v) semi-solid TSA with 10% (w/v) NaCl. Gram reactions were determined using 3% KOH (Powers, 1995). Catalase activity was examined for bubble production using 3% (v/v) hydrogen peroxide. Oxidase activity was tested by applying 1% (w/v) tetramethyl-*p*-phenylenediamine (bioMérieux, France) onto the colonies on filter paper (Kovacs, 1956). A positive reaction resulted in dark purple colonies within 5–10 s. Lactose fermentation ability was assessed on MacConkey agar (Becton, Dickinson and Company) with 10% (w/v) NaCl. Anaerobic growth was examined on TSA with 10% (w/v) NaCl under anaerobic conditions at 30°C for 20 days in an anaerobic chamber (Bactron 300; Sheldon Manufacturing, Inc., OH, United States) with an atmosphere of N_2_/CO_2_/H_2_ (90:5:5, v/v/v). The ability to perform anaerobic respiration using nitrate or nitrite as an alternative electron acceptor was determined by adding 0.2 g/L of potassium nitrate or potassium nitrite to TSB with 10% (w/v) NaCl medium under anaerobic conditions, respectively.

### 2.6 Biochemical characterization

Metabolic activity was assessed using Biolog GEN III MicroPlate™ (Biolog Inc., CA, United States) containing 71 carbon sources and 23 chemical sensitivities. Cell suspensions were inoculated and incubated at 30°C for 24 h. Plates were monitored using a Biolog Microstation (Biolog Inc.) at a wavelength of 590 nm. Biochemical features and enzyme activities were determined using API 20NE and API ZYM (bioMérieux) according to the manufacturer’s instructions. The Voges-Proskauer reaction was tested using VP medium (7 g/L peptone, 5 g/L dextrose, and 5 g/L dipotassium phosphate) containing 10% (w/v) NaCl. After incubation for 24 h, 5% (w/v) 1-naphthol and 40% (w/v) potassium hydroxide were added sequentially to the culture broth. The gluconate oxidation test was performed on the following medium: 1.5 g/L peptone; 1.0 g/L yeast extract; 1.0 g/L K_2_HPO_4_; and 40 g/L potassium gluconate. After incubation at 30°C for 48 h, Benedict’s reagent was added to the culture, and the mixture was boiled for 5–10 min. Tyrosine hydrolysis was tested on TSA supplemented with 10% (w/v) NaCl and 5 g/L tyrosine by observing a clear zone of disappearance of tyrosine crystals (Gordon and Smith, 1955).

### 2.7 Chemotaxonomic characterization

#### 2.7.1 Polar lipid

Polar lipids were extracted from strains SG2L-4^T^, SB1M-4, and SB2L-5, as described by Minnikin et al. (1977) and subsequently analyzed using thin-layer chromatography (TLC). TLC Silica gel 60 F_254_ plates (aluminum sheet, 10 cm × 10 cm; Merck, Germany) were developed with a solvent comprising chloroform:methanol:water (65:25:4, v/v) for one-dimensional chromatography and chloroform:acetic acid:methanol:water (80,15:12:4, v/v) for two-dimensional chromatography. Plates were then sprayed with the following reagents to detect different types of polar lipids: 10% (w/v) molybdophosphoric acid for total lipids; 0.2% (v/v) ninhydrin for lipids with free amino groups; molybdenum blue reagent for phospholipids; and *ɑ*-naphthol-sulfuric acid for glycolipids.

#### 2.7.2 Respiratory quinone

Respiratory quinone was extracted from freeze-dried cells using chloroform:methanol (2:1, v/v) according to a previously described method (Collins and Jones, 1981). Quinone was separated and analyzed using an HPLC system (Shimadzu Corp., Japan) equipped with a CBM-20A communication bus module, DGU-20A degassing unit, LC-20 AD pumps, a CTO-20A column oven, and an SPD-20A UV/VIS detector. The extracted quinone samples were isocratically eluted on ZORBAX SB-C18 ODS column (4.6 mm × 250 mm, 5 μm; Agilent Technologies, CA, United States) with degassed methanol:diisopropyl ether (3:1, v/v) as the eluent at a flow rate of 1 mL/min. Ubiquinone was detected at a UV detection wavelength of 275 nm. The quinone from *H. garicola* KACC 18117^T^, known to contain ubiquinone 9 (Q-9), was analyzed as a standard to compare retention times. All samples were filtered through a 0.45 μm Advantech membrane filter.

#### 2.7.3 Cellular fatty acid

Cellular fatty acid methyl esters were isolated, saponified, methylated, extracted, and washed according to the standard MIDI protocol (Sasser, 1990). Fatty acids were detected using gas chromatography–mass spectrometry and identified using the Microbial Identification Sherlock software package with the aerobic database version 6.1.

### 2.8 Examination of EPS production

To confirm EPS production, the novel strains were grown on a modified MY agar medium (10 g/L glucose, 3 g/L yeast extract, 3 g/L malt extract, 5 g/L peptone, and 100 g/L NaCl) at 30°C for 7 days (Quesada et al., 1993) and underwent SEM analysis. To obtain EPS from strains SG2L-4^T^, SB1M-4, and SB2L-5, broth cultures incubated at 150 rpm at 30°C for 7 days were centrifuged at 7,000 rpm for 10 min. The cell-free supernatant was mixed with three volumes of cold absolute ethanol and kept at 4°C overnight. Subsequently, 4% (w/v) trichloroacetic acid was added to the pellet after centrifugation and stored at 4°C for 2 h to induce protein precipitation. After removing the protein precipitate, the remaining soluble protein was filtered through a 0.2 μm membrane filter. The obtained supernatant was treated with two volumes of cold absolute ethanol overnight at 4°C to separate the precipitate, which was then dried. The dried sample was dissolved in hot distilled water, dialyzed at 4°C for 24 h using dialysis tubing (MW cut off 12–14 kDa, Thermo Fisher Scientific), and lyophilized. Crude EPS yield was measured, and the presence of carbohydrates was determined by Molisch’s test using glucose as the standard (Teekanam et al., 2023).

## 3 Results and discussion

### 3.1 Phenotypic and biochemical characteristics

**Figure 1:**
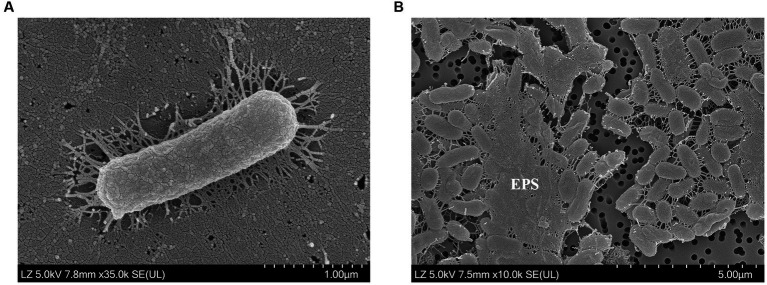
Scanning electron microscopy images of **(A)** cell morphology and **(B)** exopolysaccharides (EPS) produced by *Halomonas piscis* SG2L-4^T^. The strain was incubated on **(A)** TSA with 10% (w/v) NaCl at 30°C for 2 days and **(B)** MY medium at 30°C for 7 days.

Strains SG2L-4^T^, SB1M-4, and SB2L-5 were Gram-stain-negative, oxidase-negative, catalase-positive, non-motile, and rod-shaped cells without flagella (Figure 1A). Colonies on TSA supplemented with 10% (w/v) NaCl appeared creamy-beige or creamy-pink, circular with entire margins (<1 mm) after 48 h incubation at 30°C. Strain SG2L-4^T^ grew at temperatures between 10 and 37°C (optimum 30–37°C), at pH levels of 6–9 (optimum pH 7), and in the presence of 4–30% (w/v) NaCl (optimum 10%). Strains SB1M-4 and SB2L-5 grew in the temperature range of 10–37°C and at pH values of 6–8, with NaCl tolerance between 3 and 30%. None of the strains displayed anaerobic growth on TSA supplemented with 10% (w/v) NaCl. Strains SG2L-4^T^ and SB2L-5 were capable of anaerobic respiration when nitrite was used as the terminal electron acceptor, whereas SB1M-4 was not. Furthermore, among the three strains, only SB2L-5 employed nitrate for respiration.

All the isolates grew on MacConkey agar containing 10% (w/v) NaCl. Strains SG2L-4^T^, SB1M-4, and SB2L-5 were negative for the Voges-Proskauer test and positive for gluconate oxidation. Strains SG2L-4^T^ and SB1M-4, but not SB2L-5, produced tyrosine pigments. All strains were negative for D-glucose oxidation or fermentation. Biolog GENIII microplate assay revealed that strain SG2L-4^T^ utilized L-fucose, D-fructose-6-PO_4_, D-aspartic acid, glycyl-L-proline, L-alanine, L-aspartic acid, L-glutamic acid, L-histidine, L-serine, D-galacturonic acid, D-galactonic acid lactone, D-glucuronic acid, glucuronamide, methyl pyruvate, D-lactic acid methyl ester, L-lactic acid, L-malic acid, bromo-succinic acid, *ɑ*-hydroxy-butyric acid, *ɑ*-keto-butyric acid, acetoacetic acid, propionic acid, and acetic acid. API 20NE test strip results indicated that strains SG2L-4^T^, SB1M-4, and SB2L-5 were negative for L-tryptophane, D-glucose, L-arginine, urea, esculin ferric citrate, gelatin, 4-nitrophenyl-*β*-D-galactopyranoside, glucose assimilation, L-arabinose, D-mannose, D-mannitol, N-acetyl-glucosamine, D-maltose, potassium gluconate, capric acid, adipic acid, malic acid, trisodium citrate, and phenylacetic acid. The reduction of nitrate to nitrite was positive for strains SG2L-4^T^ and SB2L-5 but negative for strain SB1M-4. In the API ZYM test strip, strains SG2L-4^T^, SB1M-4, and SB2L-5 were positive for esterase (C4), esterase lipase (C8), leucine arylamidase, valine arylamidase, and naphthol-AS-BI-phosphohydrolase; and negative for alkaline phosphatase, lipase (C14), cystine arylamidase, trypsin, *ɑ*-chymotrypsin, acid phosphatase, naphthol-AS-BI-phosphohydrolase, *ɑ*-galactosidase, *β*-glucuronidase, *β*-glucosidase, *ɑ*-glucosidase, N-acetyl-*β*-glucosaminidase, *ɑ*-mannosidase, and *ɑ*-fucosidase.

The physiological and biochemical characteristics distinguishing strains SG2L-4^T^, SB1M-4, and SB2L-5 from their closely related type strains are summarized in Tables 1, 2.

**Table 1:** Differential phenotypic characteristics of strains SG2L-4^T^, SB1M-4, SG2L-5, and the closely related *Halomonas* species.

Characteristic	1	2	3	4	5	6
Origin^*^	Yellow corvina jeotgal	Yellow corvina jeotgal	Mixed jeotgal	Shrimp jeotgal	Jeotgal	Shrimp jeotgal
Colony color^*^	Creamy-pink	Cream beige	Cream beige	Pale-yellow	Cream	Creamy-pink
Motility^*^	−	−	−	−	−	+
Flagellation^*^	Absent	Absent	Absent	Absent	Absent	Peritrichous
Oxidase	−	−	−	−	+	+
Temperature range (°C)^*^	10–37	10–37	10–37	20–37	10–32	15–35
pH range^*^	6–9	6–8	6–8	5.5–9.5	5–10	5.5–9.0
Salt range (%, w/v)^*^	4–30	3–30	4–30	3–22.5	5–25	3–15
Growth on MacConkey agar	+	+	+	+	−	+
Anaerobic growth on MH agar^*^	−	−	−	−	−	+
Oxidation or fermentation of D-glucose^*^	−	−	−	−	Oxidative	−
Gluconate oxidation^*^	+	+	+	−	+	−
Tyrosine pigment production	+	+	−	−	−	−
Voges-Proskauer test^*^	−	−	−	+	−	−
Respiration with nitrate	−	−	+	+	−	+
Respiration with nitrite	+	−	+	−	−	+

Strains: 1, SG2L-4^T^; 2, SB1M-4; 3, SB2L-5; 4, *H. garicola* KACC 18117^T^; 5, *H. jeotgali* KCTC 22487^T^; 6, *H. cibimaris* KACC 14932^T^. +, positive; −, negative. ^*^Data are from Jung et al. (2016) for *H. garicola* KACC 18117^T^, Kim et al. (2010) for *H. jeotgali* KCTC 22487^T^, and Jeong et al. (2013) for *H. cibimaris* KACC 14932^T^.

**Table 2:** Differential biochemical characteristics between strains SG2L-4^T^, SB1M-4, SG2L-5, and their closely related type strains.

Characteristic	1	2	3	4	5	6
*Utilization of*:						
Gentiobiose, D-turanose, D-melibiose, N-acetyl-*β*-D-mannosamine, *α*-D-glucose, D-fructose, D-galactose, 3-methyl glucose, L-rhamnose, D-glucose-6-PO_4_, L-arginine, L-histidine, D-gluconic acid, citric acid, *α*-keto-glutaric acid, tween 40, *β*-hydroxy-D,L-butyric acid	−	−	−	−	+	−
D-Fructose-6-PO_4_, L-lactic acid, L-malic acid, bromo-succinic acid	+	+	+	+	−	+
L-Fucose, acetoacetic acid	+	+	+	−	+	+
L-Serine, *α*-hydroxy-butyric acid	+	−	+	+	+	+
Dextrin, D-serine	−	−	−	−	−	+
D-Fucose	−	+	−	−	+	−
D-Aspartic acid	+	−	+	+	−	+
Methyl pyruvate	+	−	−	+	−	+
D-Lactic acid methyl ester	+	+	−	+	+	+
D-Malic acid	−	−	−	+	−	−
*γ*-Amino-butyric acid	−	−	+	−	+	−
*α*-Keto-butyric acid	+	−	−	+	−	−
Formic acid	−	−	+	+	−	−

Strains: 1, SG2L-4^T^; 2, SB1M-4; 3, SB2L-5; 4, *H. garicola* KACC 18117^T^; 5, *H. jeotgali* KCTC 22487^T^; 6, *H. cibimaris* KACC 14932^T^. +, positive; −, negative.

### 3.2 Chemotaxonomic characteristics

Chemotaxonomic analysis revealed that the major ubiquinone in strains SG2L-4^T^, SB1M-4, and SB2L-5 was Q-9, consistent with other *Halomonas* species (Supplementary Figure S1). Polar lipid profiles in the novel strains included diphosphatidylglycerol (DPG), phosphatidylethanolamine (PE), phosphatidylglycerol (PG), an unknown phospholipid (PL), an unknown glycolipid (GL), and an unknown polar lipid (L) (Supplementary Figure S2). These profiles resembled those of closely related strains (Kim et al., 2010; Jeong et al., 2013; Jung et al., 2016). The predominant (>10%) cellular fatty acids in strain SG2L-4^T^ were C_16:0_ (28.4%), summed features 8 (C_18:1_ *ω*6*c* and/or C_18:1_ *ω*7*c*; 24.5%), C_19:0_ cyclo *ω*8*c* (16.5%), and summed features 3 (C_16:1_ *ω*6*c* and/or C_16:1_ *ω*7*c*; 10.7%). The complete composition of cellular fatty acids for strains SG2L-4^T^, SB1M-4, and SB2L-5, and the reference strains is provided in Table 3.

**Table 3:** Cellular fatty acid content of strains SG2L-4^T^, SB1M-4, SB2L-5, and the related species of the genus *Halomonas.*

	1	2	3	4	5	6
*Saturated fatty acids*						
C_10:0_	2.2	2.3	2.4	2.2	2.9	1.2
C_12:0_	3.4	4.4	4.1	3.5	3.8	2.0
C_14:0_	0.6	0.9	0.7	0.7	–	–
C_16:0_	**28.4**	**29.7**	**27.8**	**28.4**	**29.1**	**24.1**
C_18:0_	1.0	tr	3.6	1.7	tr	tr
*Hydroxy fatty acids*						
C_12:0_ 3-OH	6.7	9.6	8.3	5.6	9.6	7.6
*Unsaturated fatty acids*						
C_18:1_ *ω*9*c*	–	–	tr	1.0	–	–
*Cyclo fatty acid*						
C_17:0_ cyclo	4.8	2.1	2.8	4.5	–	–
C_19:0_ cyclo *ω*8*c*	**16.5**	8.5	**11.4**	**13.1**	tr	–
*Summed features* ^*^						
3	**10.7**	**14.5**	**12.6**	**14.0**	8.3	**10.3**
8	**24.5**	**26.5**	**24.1**	**24.6**	**43.3**	**52.8**

Strains: 1, SG2L-4^T^; 2, SB1M-4; 3, SB2L-5; 4, *H. garicola* KACC 18117^T^; 5, *H. jeotgali* KCTC 22487^T^; 6, *H. cibimaris* KACC 14932^T^. All data were obtained from this study. Data are expressed as percentages of the total fatty acids. Major components (>10%) are highlighted in bold. Fatty acids constituting <0.5% of the total content in all strains are not displayed. tr, trace amount (<1%); −, not detected. ^*^Summed features represent groups of two or three fatty acids that could not be separated via GLC using the MIDI system. Summed feature 3 contains C_16:1_ ω6c and/or C_16:1_ ω7c; summed feature 8 contains C_18:1_ ω6c and/or C_18:1_ ω7c.

### 3.3 The 16S rRNA gene sequences and phylogenetic analyses

**Figure 2:**
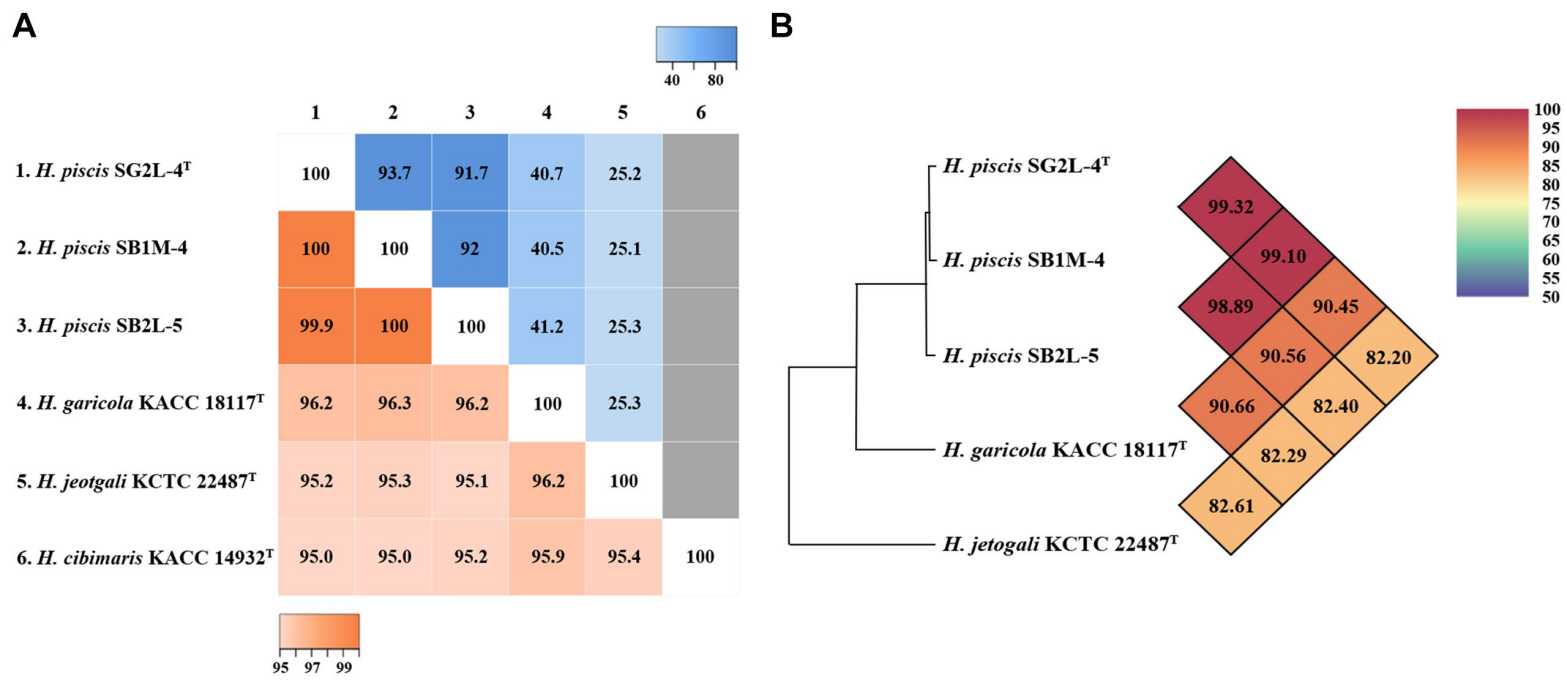
Genomic relatedness of the novel strains and closely related *Halomonas* species. **(A)** Heatmap of pairwise similarity based on 16S rRNA gene sequences (lower left), dDDH values based on whole genome sequence (upper right), and **(B)** OrthoANI values between strains SG2L-4^T^, SB1M-4, SB2L-5, and closely related *Halomonas* species. The pairwise similarity and dDDH values are visualized using Heatmapper tool. The gray color represents the absence of the genome sequence for *H. cibimaris* KACC 14932^T^. OrthoANI values calculated using OAT software.

**Figure 3:**
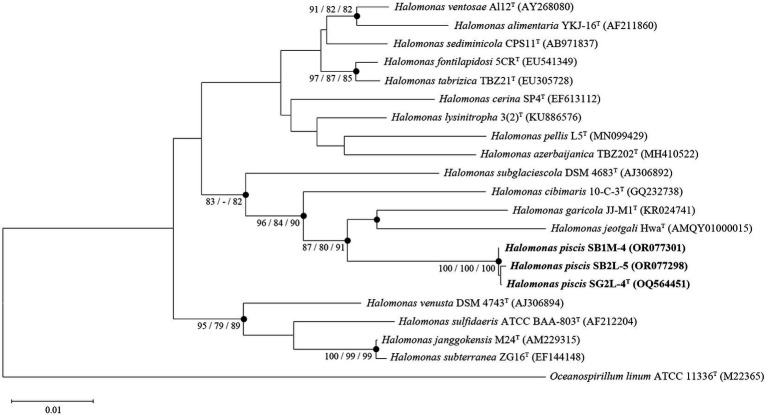
A phylogenetic consensus tree based on 16S rRNA gene sequences, reconstructed using the neighbor-joining (NJ), maximum-parsimony (MP), and maximum-likelihood (ML) algorithms. The filled circles indicate the corresponding branches generated by all three algorithms. Node numbers represent bootstrap values (NJ/MP/ML) as a percentage of 1,000 replicates. Values higher than 70% are shown at the branch points. *Oceanospirillum linum* ATCC 11336^T^ served as an outgroup. Scale bar: 0.01 accumulated changes per nucleotide position.

Analysis of 16S rRNA gene sequences revealed that strains SG2L-4^T^, SB1M-4, and SB2L-5 shared the highest similarity with *H. garicola* KACC 18117^T^ (96.2–96.3%) (Figure 2A). The pairwise similarity of strain SG2L-4^T^ to strains SB1M-4 and SB2L-5 was 100 and 99.9% (1 bp difference), respectively. The phylogenetic tree constructed based on 16S rRNA gene sequences showed that strains SG2L-4^T^, SB1M-4, and SB2L-5 belonged to the genus *Halomonas* and formed a cluster with *H. garicola* KACC 18117^T^, *H. jeotgali* KCTC 22487^T^, and *H. cibimaris* KACC 14932^T^ (Figure 3). Based on 16S rRNA gene sequence similarity and phylogeny, *H. garicola* KACC 18117^T^, *H. jeotgali* KCTC 22487^T^, and *H. cibimaris* KACC 14932^T^ were selected as reference species for further comparison.

### 3.4 Genomic attributes and relatedness indices

The phylogenomic tree based on the whole-genome sequences showed that strains SG2L-4^T^, SB1M-4, and SB2L-5 clustered with *H. garicola* KACC 18117^T^ and *H. cibimaris* KACC 14932^T^, consistent with 16S rRNA gene sequence-based phylogenetic tree (Supplementary Figure S3). To assess the genetic relatedness between the isolated strains and their closest phylogenetic relatives, ANI and dDDH values were calculated (Figure 2). Strains SG2L-4^T^, SB1M-4, and SB2L-5 exhibited ANI values of 90.45, 90.56, and 90.66%, respectively, in comparison to *H. garicola* KACC 18117^T^, with corresponding dDDH values of 40.7, 40.5, and 41.2%. According to previous studies, the generally accepted thresholds for delineating novel species in bacterial taxonomy have been defined as ≤95–96% for ANI values (Maderankova et al., 2019) and ≤70% for dDDH values (Auch et al., 2010) when compared to their closest related species. Based on phylogenomic analysis and genomic relatedness indices, strains SG2L-4^T^, SB1M-4, and SB2L-5, named *Halomonas piscis* sp. nov., represent novel species belonging to the genus *Halomonas.*

The general genomic features of strains SG2L-4^T^, SB1M-4, and SB2L-5 and the reference strains are summarized in Table 4. Strain SG2L-4^T^ had a single circular chromosome of 3,227,066 bp in size, comprising a single contig containing 3,135 genes, including 3,055 protein-coding genes (CDS), 62 tRNA genes, and 18 rRNA genes. The estimated genome sizes of the strains SB1M-4 and SB2L-5 were 3,295,832 bp and 3,385,314 bp, respectively. The genome of strain SB1M-4 was assembled into 135 contigs with 2,871 CDS, 57 tRNA, and 6 rRNA genes, whereas that of strain SB2L-5 comprised 156 contigs, including 3,095 CDS, 61 tRNA, and 6 rRNA genes. The genomic G + C content of strains SG2L-4^T^, SB1M-4, and SB2L-5 was 63.3, 64.0, and 63.3 mol%, respectively, similar to that of the closely related strains (61.7–63.7 mol%). Annotated functional information for strains SG2L-4^T^, SB1M-4, SB2L-5, and the reference strains is provided in Supplementary Figure S4, allowing for a comparison of similarities and peculiarities. Strain SG2L-4^T^ had 2,921 coding sequences and 299 subsystems, with the most represented subsystem features being “Amino Acid and Derivatives,” “Protein metabolism,” “Cofactors, Vitamins, Prosthetic Groups, Pigments,” and “Carbohydrates.” Strain SG2L-4^T^ exhibited enrichment in genes associated with protein and RNA metabolism compared to other strains. These features maintain cellular functions, and adapt to environmental changes, contributing to cell health and survival (Anantharaman et al., 2002). In addition, their diversity enhances organismal functionality and adaptability, playing crucial roles in cell signaling and development processes.

**Table 4:** Comparison of general genomic features between strains SG2L-4^T^, SB1M-4, SB2L-5, and the reference strains.

	1	2	3	4	5
Genome size (bp)	3,227,066	3,295,832	3,385,314	2,933,783	2,847,098
Number of contigs	1	135	156	73	26
G + C content (%)	63.3	64.0	63.3	63.7	61.7
N_50_	3,227,066	43,403	41,472	70,100	236,076
Genes	3,135	2,871	3,095	2,741	2,683
Number of CDS^*^	3,055	2,808	3,028	2,680	2,613
Number of tRNA^*^ genes	62	57	61	57	58
Number of rRNA^*^ genes	18	6	6	4	12
Accession number	CP119391	JASSPR000000000	JASSPS000000000	JASSZF000000000	GCF 000334215

Strains: 1, SG2L-4^T^; 2, SB1M-4; 3, SB2L-5; 4, *H. garicola* KACC 18117^T^; and 5, *H. jeotgali* KCTC 22487^T^. ^*^CDS, protein-coding genes; tRNA, transfer RNA; rRNA, ribosomal RNA.

**Figure 4:**
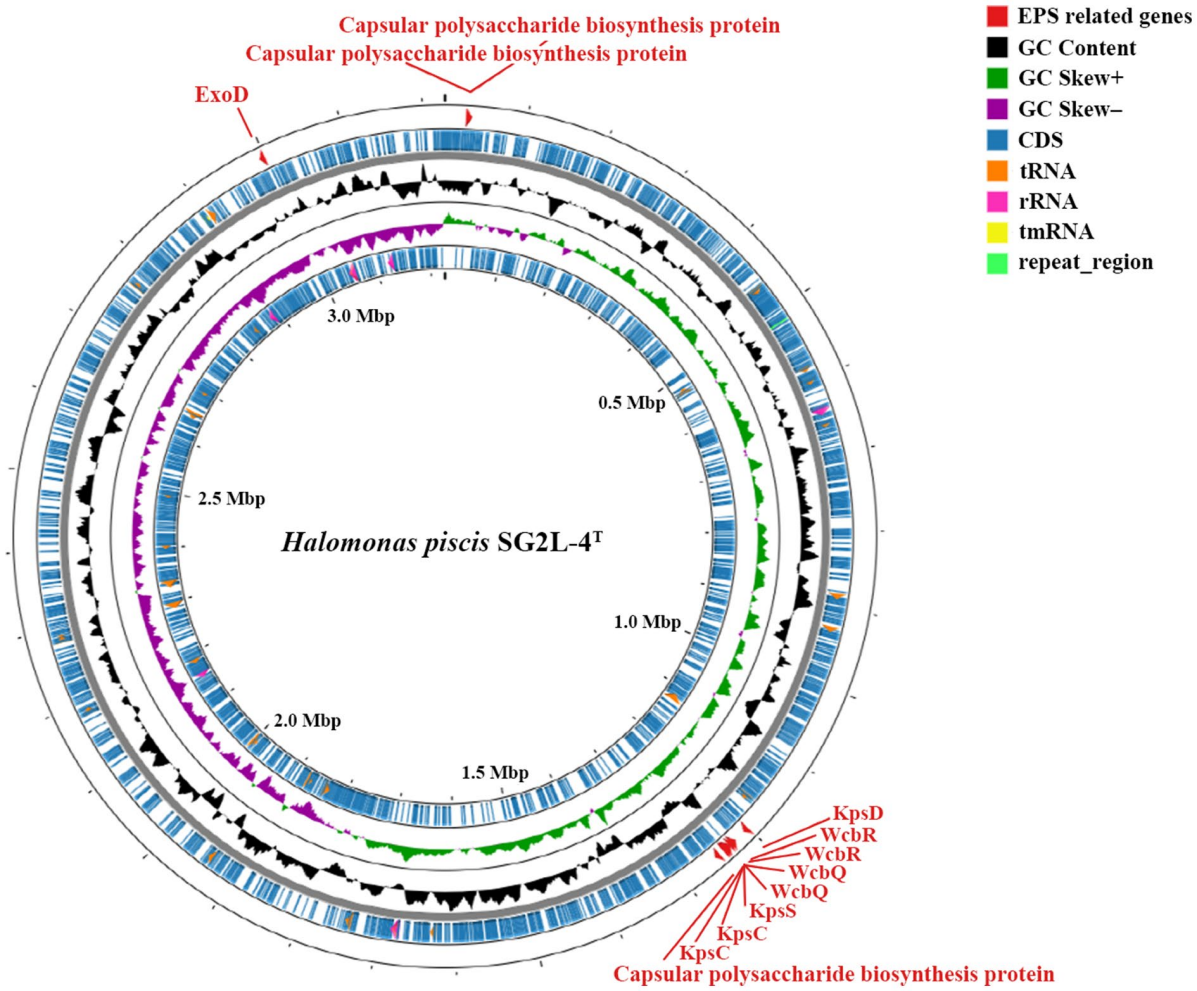
Circular genome map of *Halomonas piscis* SG2L-4^T^ generated using CGView server. The gray ring is the backbone of the sequence. The feature ring indicates the distribution of EPS-related genes (red), protein-coding genes (CDS; blue), GC content (black), GC skew (green and violet), tRNA genes (orange), rRNA genes (pink), tmRNA genes (yellow), and repeat region (light green).

An annotated circular map including the genomic features of strain SG2L-4^T^ is shown in Figure 4. The outermost ring of the circular map highlights the presence of EPS synthesis-related gene, ExoD, which contributes to the formation of a protective film on the microbial cell surface. Similarly, it was observed that the reference strain *H. garicola* KACC 18117^T^ possesses both ExoD and capsular polysaccharide genes, whereas these genes were notably absent in *H. jeotgali* KCTC 22487^T^. This finding demonstrates the EPS production capability of the strain SG2L-4^T^ and suggests that the produced EPS can be utilized environmentally friendly and economically in various applications as a potential carbon source (Notararigo et al., 2013; Raghavan et al., 2023). In addition, genomic analysis of the strain SG2L-4^T^ revealed the presence of genes associated with capsular polysaccharide production, which forms a thick protective layer on the exterior of the microorganisms, preventing microbial attachment to the surface. KpsC, KpsD, and KpsS are responsible for encoding enzymes and proteins involved in the biosynthesis and export of capsular polysaccharides (Karlyshev et al., 2000). KpsC generates key components of the outer polysaccharide layer, whereas KpsD and KpsS contribute to the generation of polysaccharide units and their assembly into the outer layer. The function of these genes can vary among different bacterial species and strains, but they are essential for determining the composition and structural characteristics of the capsular polysaccharides. Additionally, the presence of WcbQ and WcbR genes in the genome indicates the involvement of SG2L-4^T^ in capsular polysaccharide biosynthesis (Warawa et al., 2009). These genome annotation results suggest the potential EPS-producing capability of *H. piscis* sp. nov. SG2L-4^T^.

### 3.5 Production of exopolysaccharides

SEM analysis revealed the presence of overlaid microstructured EPS in strain SG2L-4^T^ (Figure 1B). To confirm EPS production in strains SG2L-4^T^, SB1M-4, and SB2L-5, Molisch’s test was conducted according to the method of Sudhesh and Sumathy (2016). A violet ring was observed in the cultures of the novel strains, indicating the presence of extracellular carbohydrates (Supplementary Figure S5). *H. vantosae* and *H. anticariensis* were reported to secrete 283.5 and 289.5 mg/L of EPS, respectively (Mata et al., 2006). Notably, the strains SG2L-4^T^, SB1M-4, and SB2L-5 exhibited substantial EPS production, producing 420, 320, and 480 mg/L of crude EPS, respectively. These findings not only validate the EPS-producing potential of these isolated strains but also highlight their remarkable capability, surpassing previously reported *Halomonas* species in terms of yield.

Microbial-derived EPS has gained prominence for commercial applications in various industries including food, agriculture, and pharmaceuticals (Küçükaşik et al., 2011; Srivastava and Sharma, 2023). For instance, crude EPS derived from *Phellinus baumii* exhibited anti-diabetic effects in streptozotocin-induced diabetes (Hwang et al., 2005). EPS produced by acetic acid-producing bacteria improved the rheological properties of fermented foods, and the addition of EPS derived from *Lactobacillus plantarum* to cheese improves the moisture content, proteolysis, and textural properties, increasing cheese yield (Broadbent et al., 2001). Starter cultures such as *L. delbruecki* subsp. *bulgaricus* and *Streptococcus thermophiles*, which yielded 60–150 and 30–890 mg/L of EPS, respectively, improved the texture, consistency, and flavor of the final yogurt product (Jurášková et al., 2022). Although the effect of these novel strains on jeotgal fermentation is unclear, previous studies have suggested that *Halomonas*-derived EPS could exert beneficial effects on the taste and flavor of fermented foods such as jeotgal. Consequently, this study emphasizes the potential of these novel strains as natural food additives. Further research is needed to understand the effects of crude EPS produced by *H. piscis* sp. nov. during jeotgal ripening.

## 4 Conclusion

Three Gram-stain-negative halophilic bacterial strains, designated SG2L-4^T^, SB1M-4, and SB2L-5, were isolated from jeotgal. Based on phylogenetic, genomic, phenotypic, and chemotaxonomic characteristics, these strains were identified as a novel species belonging to the genus *Halomonas*, for which the name *Halomonas piscis* sp. nov. is proposed. Genomic annotation of the novel type strain exhibited the presence of EPS biosynthesis-related genes, including ExoD (exopolysaccharide synthesis), KpsD (capsular polysaccharide export system periplasmic protein), KpsS (capsular polysaccharide export system protein), KpsC (capsular polysaccharide export system protein), WcbR (capsular polysaccharide biosynthesis fatty acid synthase), and WcbQ (capsular polysaccharide biosynthesis protein), in the whole-genome sequence. Furthermore, the EPS production capability of the novel strains was confirmed by detecting extracellular carbohydrates using Molisch’s test. Our results suggest the potential of *H. piscis* sp. nov. as a novel candidate for the application of microbial EPS in various fields.

### 4.1 Description of *Halomonas piscis* sp. nov.

#### 4.1.1 *Halomonas piscis* (pis’cis. L. gen. *Piscis*, of a fish)

Cells are Gram-stain-negative, aerobic, non-motile, positive for catalase, negative for oxidase, and rod-shaped (0.6–0.8 μm in width and 1.8–9.7 μm in length) without flagella. The optimal growth occurs at temperatures between 30 and 37°C, pH value between 7 and 8, and in the presence of 7–10% (w/v) NaCl. Colonies are creamy-pink or creamy-beige and circular (<1 mm) with entire margins, after incubation at 30°C for 2 days on TSA with 10% (w/v) NaCl. Growth was observed on MacConkey agar supplemented with 10% (w/v) NaCl. Anaerobic growth was not observed. Anaerobic respiration using nitrate or nitrite was observed. Positive for gluconate oxidation and tyrosine pigment production tests, while negative for Voges-Proskauer test and hydrolysis of asculin. In the API ZYM test, positive for esterase (C4), esterase lipase (C8), leucine arylamidase, valine arylamidase, and naphthol-AS-BI-phosphohydrolase but negative for other substrates. On the API 20NE strip, only the reduction of nitrates to nitrites resulted in a positive reaction. In the Biolog GEN III MicroPlate™ test, the results are positive for D-fucose, L-fucose, D-fructose-6-PO_4_, D-aspartic acid, glycyl-L-proline, L-alanine, L-aspartic acid, L-glutamic acid, L-histidine, L-serine, D-galacturonic acid, D-galactonic acid lactone, D-glucuronic acid, glucuronamide, methyl pyruvate, D-lactic acid methyl ester, L-lactic acid, L-malic acid, bromo-succinic acid, *ɑ*-hydroxy-butyric acid, *ɑ*-keto-butyric acid, acetoacetic acid, propionic acid, acetic acid, and formic acid. Q-9 is the sole respiratory quinone. The polar lipids included DPG, PE, PG, PL, GL, and L. The predominant cellular fatty acids are C_16:0_, summed features 8 (C_18:1_ *ω*6*c* and/or C_18:1_ *ω*7*c*), C_19:0_ cyclo *ω*8*c*, and summed features 3 (C_16:1_ *ω*6*c* and/or C_16:1_ *ω*7*c*).

The type strain SG2L-4^T^ (=KCTC 92842^T^ = JCM 35929^T^) was isolated from yellow corvina jeotgal from a Korean traditional market in the Republic of Korea. The genomic G + C content of the type strain was 63.3 mol%. The GeneBank accession numbers for the 16S rRNA gene and whole-genome sequence of the type strain are OQ564451 and CP119391, respectively.

## Data availability statement

The datasets presented in this study can be found in online repositories. The names of the repository/repositories and accession number(s) can be found in the article/Supplementary material.

## Author contributions

BK: Conceptualization, Formal analysis, Investigation, Methodology, Validation, Visualization, Writing – original draft, Writing – review & editing. A-IY: Formal analysis, Investigation, Writing – review & editing. H-IJ: Formal analysis, Writing – review & editing. KK: Formal analysis, Writing – review & editing. HC: Formal analysis, Writing – review & editing. S-HJ: Formal analysis, Writing – review & editing. MJ: Formal analysis, Writing – review & editing. N-RS: Conceptualization, Data curation, Funding acquisition, Project administration, Resources, Supervision, Validation, Writing – review & editing.

## Footnotes

^1^ http://www.ezbiocloud.net

^2^ https://www.ezbiocloud.net/tools/pairAlign

^3^ http://www.heatmapper.ca/

^4^ https://www.ezbiocloud.net/tools/orthoani

^5^ https://ggdc.dsmz.de/ggdc.php

^6^ https://rast.nmpdr.org/rast.cgi
