# Supplementary material for: Genomic attributes and characterization of novel exopolysaccharide-producing bacterium Halomonas piscis sp. nov. isolated from jeotgal
Source: Front Microbiol. 2023 Dec 14;14:1303039. doi: 10.3389/fmicb.2023.1303039 (PMC10752968; doi:10.3389/fmicb.2023.1303039)
Supplement: Supplementary file 1 [file Data_Sheet_1.docx]

Supplementary Material

Genomic attributes and characterization of novel exopolysaccharide-producing bacterium *Halomonas piscis* sp. nov. isolated from jeotgal

Bora Kim^1^, Ah-In Yang^1,2^, Hae-In Joe^2^, Ki Hyun Kim^1^, Hanna Choe^1^, Sung-Hong Joe^1,3^, Min Ok Jun^1^, and Na-Ri Shin^1*^

^1^Biological Resource Center, Korea Research Institute of Bioscience and Biotechnology, Jeongeup-si, Jeollabuk-do 56212, Republic of Korea

^2^Department of Biology, Kyung Hee University, Seoul 02447, Republic of Korea

^3^Department of Microbiology, Chonnam National University Medical School, Hwasun 58128, Republic of Korea

*** Correspondence:**Na-Ri Shin
[nrshin@kribb.re.kr](mailto:nrshin@kribb.re.kr)

## 1 Supplementary Figures


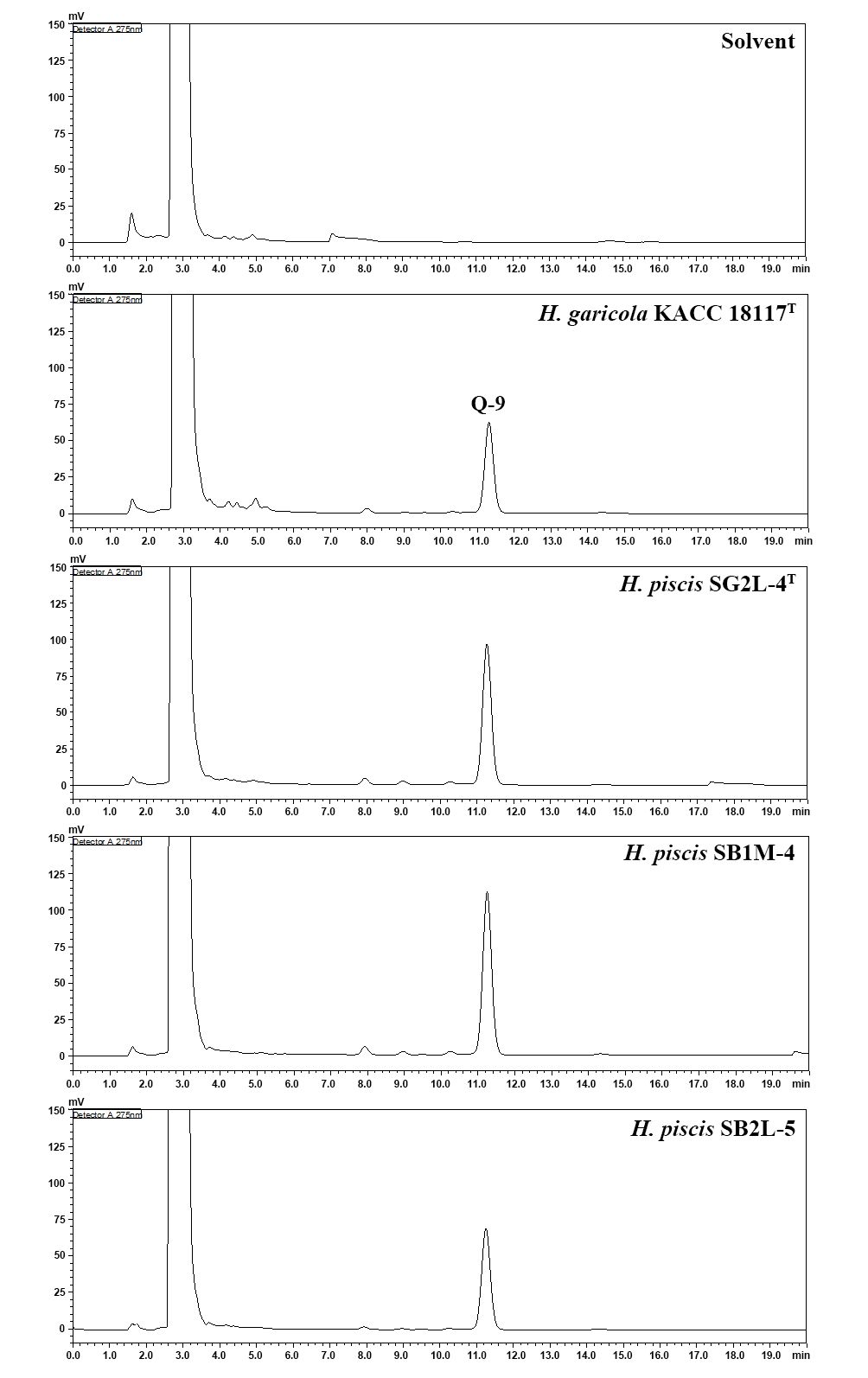


**Supplementary Figure 1.** HPLC chromatogram of the quinone compound from *Halomonas* strains. Quinone was detected by UV/Vis absorption spectroscopy at 270 nm.


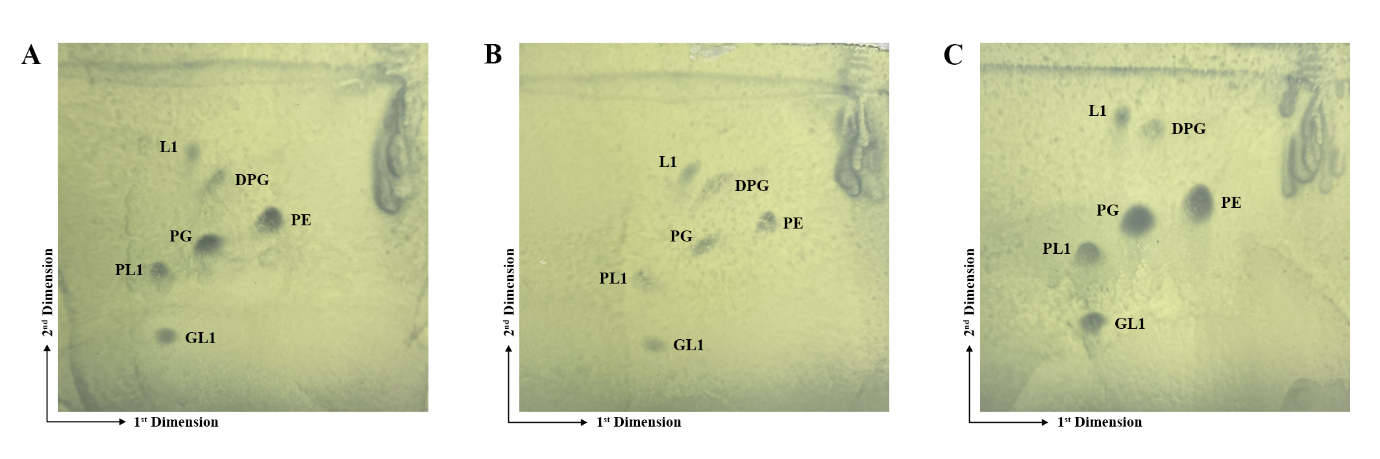


**Supplementary Figure 2.** Two-dimensional thin-layer chromatograms of polar lipids for strains SG2L-4^T^, SB1M-4, and SB2L-5. Chloroform/methanol/water (65:25:4, v/v) was used in the first direction, followed by chloroform/acetic acid/methanol/water (80:15:12:4, v/v) in the second direction. Phosphomolybdic acid solution spray was used to detect total lipids. DPG, diphosphatidylglycerol; PE, phosphatidylethanolamine; PG, phosphatidylglycerol; PL, unknown phospholipid; GL, unknown glycolipid; L, unknown polar lipid. (A) strain SG2L-4^T^, (B) strain SB1M-4, (C) strain SB2L-5.


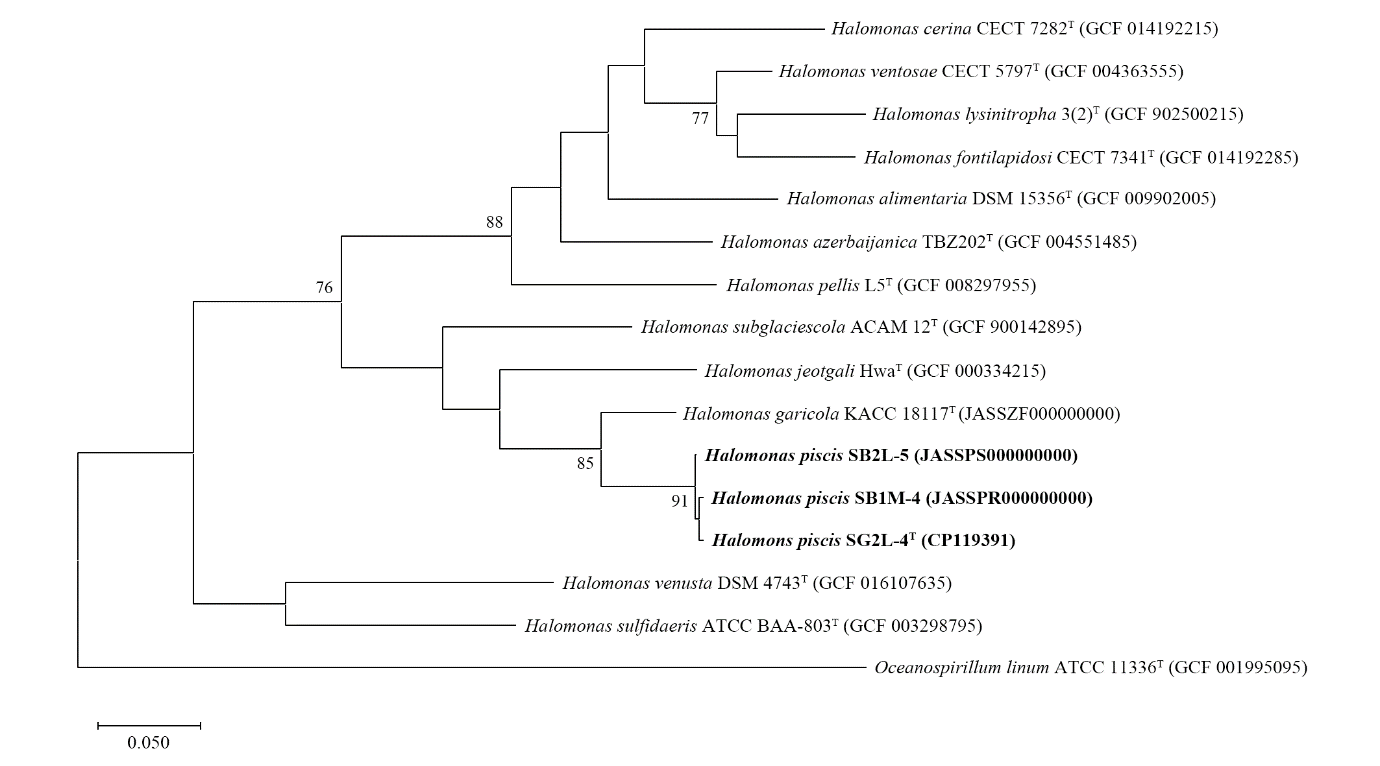


**Supplementary Figure 3.** A phylogenomic tree based on whole genome sequence. The phylogenomic tree was constructed using nearly complete genomes and represented using the UBCG with 1,000 bootstrap replicates. *Oceanospirillum linum* ATCC 11336^T^ (GCF 001995095) was used as an outgroup. Values higher than 70% are expressed at the branch points. Bar, 0.05 accumulated changes per nucleotide position.


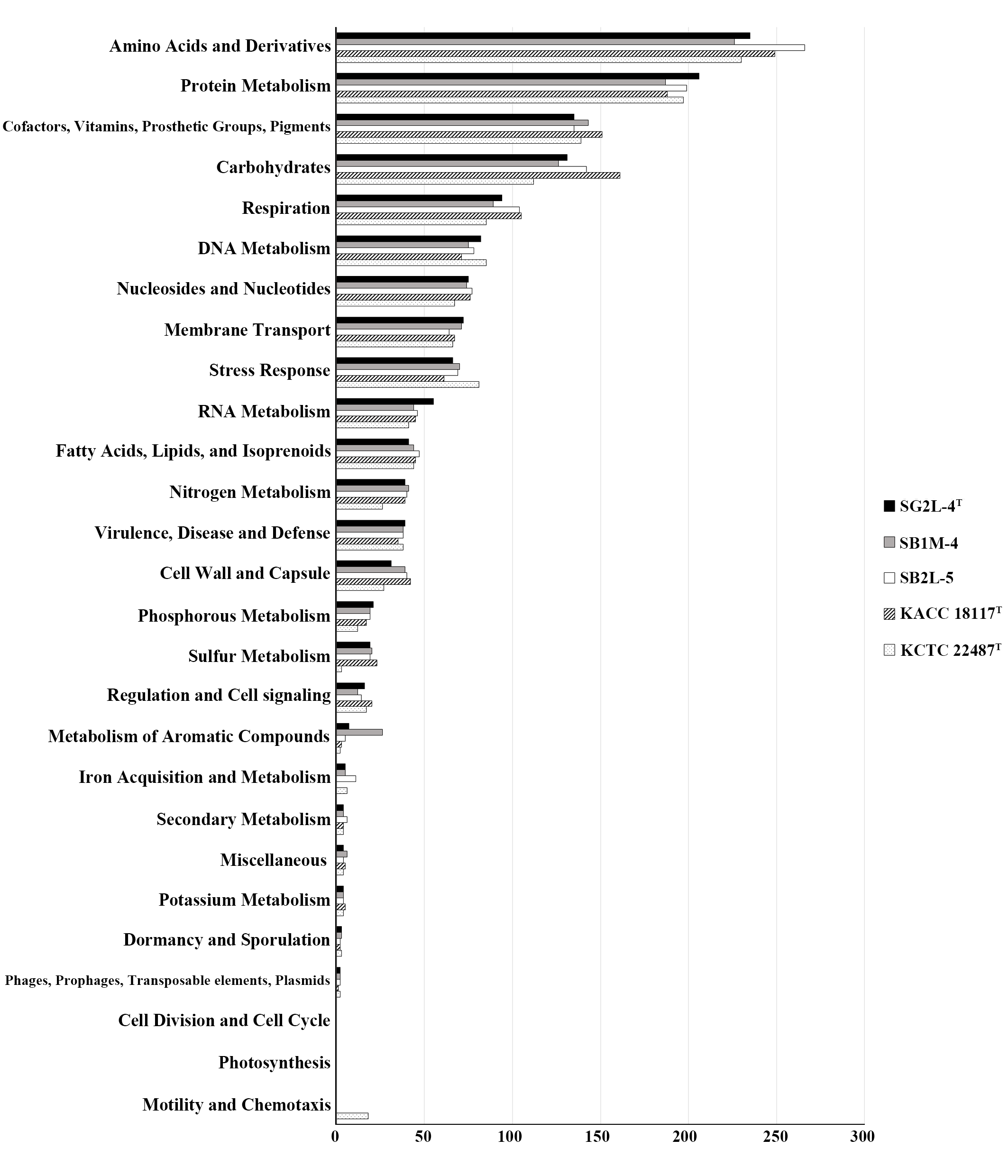


**Supplementary Figure 4.** Subsystem category distribution and features annotated to the genome of strains SG2L-4^T^, SB1M-4, SB2L-5, and the reference strains based on the RAST server.


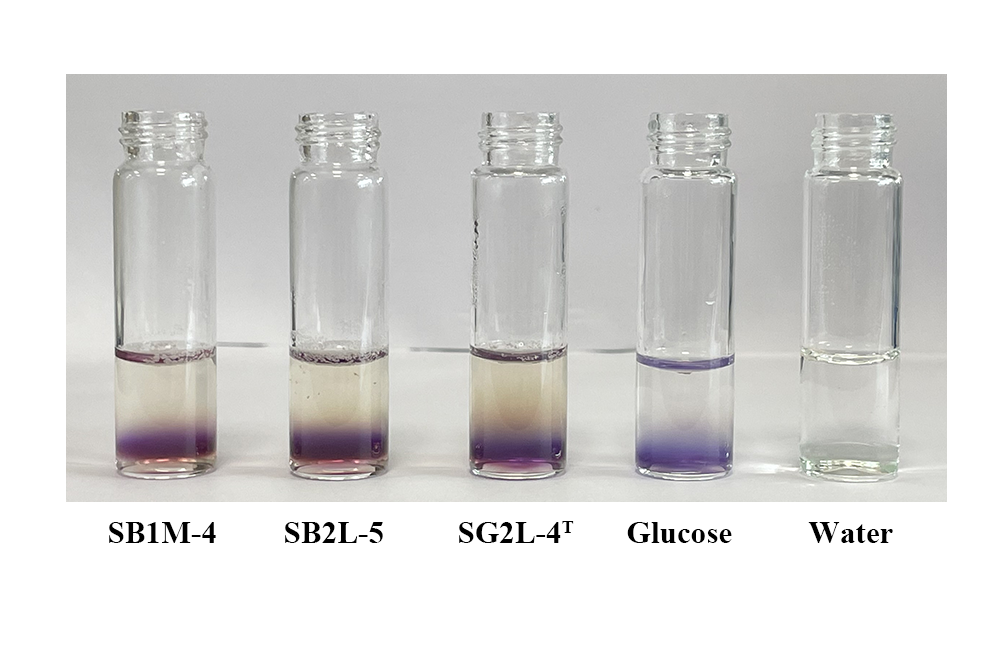


**Supplementary Figure 5.** Molisch's test for extracellular carbohydrates detection in *Halomonas piscis* strains. The appearance of a violet-colored ring between the two liquids is observed as a positive result. Glucose (1 mg/ml) and water were used as positive control and negative control, respectively.
